# An Overview of Fog Data Analytics for IoT Applications

**DOI:** 10.3390/s23010199

**Published:** 2022-12-24

**Authors:** Jitendra Bhatia, Kiran Italiya, Kuldeepsinh Jadeja, Malaram Kumhar, Uttam Chauhan, Sudeep Tanwar, Madhuri Bhavsar, Ravi Sharma, Daniela Lucia Manea, Marina Verdes, Maria Simona Raboaca

**Affiliations:** 1 Department of Computer Science and Engineering, Institute of Technology, Nirma University, Ahmedabad 382481, India; 2 Amazon, Bangalore 560055, India; 3 School of Earth and Space Exploration, Arizona State University, Tempe, AZ 85287, USA; 4 Department of Computer Engineering, Vishwakarma Government Engineering College, Gujarat Technological University, Ahmedabad 382424, India; 5 Centre for Inter-Disciplinary Research and Innovation, University of Petroleum and Energy Studies, Dehradun 248001, India; 6 Faculty of Civil Engineering, Technical University of Cluj-Napoca, Constantin Daicoviciu Street, No. 15, 400020 Cluj-Napoca, Romania; 7 Department of Building Services, Faculty of Civil Engineering and Building Services, Technical University of Gheorghe Asachi, 700050 Iași, Romania; 8 National Research and Development Institute for Cryogenic and Isotopic Technologies—ICSI Rm. Valcea, Uzinei Street, No. 4, P.O. Box 7 Raureni, 240050 Ramnicu Valcea, Romania

**Keywords:** Internet of things, artificial intelligence, blockchain, fog computing, data analytics

## Abstract

With the rapid growth in the data and processing over the cloud, it has become easier to access those data. On the other hand, it poses many technical and security challenges to the users of those provisions. Fog computing makes these technical issues manageable to some extent. Fog computing is one of the promising solutions for handling the big data produced by the IoT, which are often security-critical and time-sensitive. Massive IoT data analytics by a fog computing structure is emerging and requires extensive research for more proficient knowledge and smart decisions. Though an advancement in big data analytics is taking place, it does not consider fog data analytics. However, there are many challenges, including heterogeneity, security, accessibility, resource sharing, network communication overhead, the real-time data processing of complex data, etc. This paper explores various research challenges and their solution using the next-generation fog data analytics and IoT networks. We also performed an experimental analysis based on fog computing and cloud architecture. The result shows that fog computing outperforms the cloud in terms of network utilization and latency. Finally, the paper is concluded with future trends.

## 1. Introduction

The world is changing rapidly, and novel and disruptive paradigms are shaping our future. One such paradigm is the Internet of things (IoT), which was presented many years ago but is constantly evolving. Everything we use in our daily lives becomes more intelligent as sensors are integrated. Every device, such as radio frequency identification (RFID) tags, actuators, mobile phones, and so on, permeates our daily lives in both industrial and domiciliary fields [1]. It is projected that over 31 billion devices will be linked to the Internet by 2020, and this figure will rise further as 5G technology matures. The industry is estimated to be worth USD one trillion by 2025 [2].

IoT has now diffused into various domains that were never thought of earlier. These include healthcare, home automation, aviation, industrial automation, vehicular networks, data analytics, mobiles, smart cities, agriculture, and wearables, including watches, shoes, t-shirts, etc. [3]. However, the most prominent effect of IoT is on industrial automation, or Industry 4.0, where every device or machine is connected and communicates among themselves [4,5].

Human behavior is unpredictable and sometimes changes. Robotic systems should receive assistance from the cloud’s backend, which collects data from wearables and sensors to decide the activities that robotic systems should carry out. A robotic arm utilized in many industrial applications is controlled using an IoT interface. By capturing the movement and performing the same activities repeatedly, it minimizes human effort [6]. IoT and robotic systems are closely collaborating as Industry 4.0 develops, reshaping their relationships and enabling the creation of next-generation gadgets. The Internet of robotic things (IoRT) is a notion that results from the combination of robotic agents and the Internet of things (IoT), and it opens up new opportunities for both industrial and academic disciplines. The fourth industrial revolution’s primary outcome is creating and spreading cyber–physical systems (CPSs). Applications for CPS technology include gas distribution, transportation systems, medical equipment, and electrical power grids, among many others. In CPSs, networks interface with physical systems while conducting in-depth analysis and data extraction. IoT and CPSs provide a good framework for the growth of a new field of study called the Internet of robotic things (IoRT). In this scenario, IoRT represents the core component of robotics-integrated IoT systems, where cloud computing and networking can be implemented to accomplish elaborated tasks, enabling robots to share, network, and gather various types of information from humans and others machines [7,8].

### 1.1. Background

**Figure 1:**
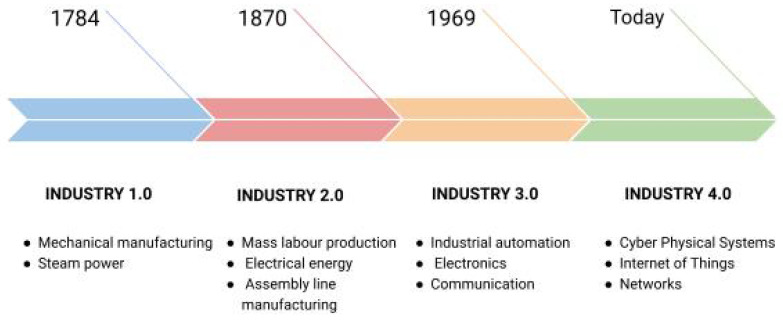
Industrial revolution.

Before we deeply understand the IoT paradigm, let us first understand the industry’s evolution through the various revolutions taking place at stipulated time intervals, each time having a novel, promising, and disruptive concept upon which that revolution has been based. Figure 1 shows the timeline of manufacturing, starting from the first industrial revolution and ending at the current state of Industry 4.0. We start with Industry 1.0, the first industrial revolution, which took place around the late 18th/early 19th century. Initially, human resources were involved in the production processes. Due to this, there was a high chance of errors, and the industries needed to be more scalable. During this revolution, the mechanization of production took place, with the introduction of steam and water-propelled engines into the industrial realm, which resulted in a tremendous increase in efficiency and scaling. Small-scale industries could now serve large companies and increase their customer base. The next revolution, Industry 2.0, took place in the 19th/early 20th century. The main factor behind the second revolution was introducing and developing machines running on electrical energy and assembly line production techniques for increasing efficiency and bringing modularization into effect so that divisions of work or labor based on skill sets could be made possible. The mass production of products began during this era. The production efficiency increased significantly due to a further decrease in human intervention as humans were now required more for monitoring and maintenance purposes and less for production. The Industry 3.0 revolution started in the late 20th century when electronic hardware such as transistors and integrated circuits were introduced to machines. These electronic devices needed software to operate them. The software also automated other tasks, including inventory management, optimum resource utilization, etc. The involvement of fully automated machines reduced the risk of errors and increased the production rate significantly. This era marked the era of computers and, lately, the Internet too.

The next and most recent industrial revolution, which is still ongoing, is the Industry 4.0 revolution, which thrives on making machines or devices “smart” [9]. It adds more value to the Industry 3.0 generation by adding communication between the connected devices/machines to minimize human involvement in production. Industry 4.0 is a byproduct of the IoT paradigm and focuses on real-time decision making by the devices based on various external and internal stimuli.

### 1.2. Introduction to IoT and Fog Computing

As we have seen in the previous section, Industry 4.0 focuses on smart devices. The devices become smart by getting equipped with sensors or actuators that help them to identify their surroundings, process the data collected from them in the brain, make a decision, and then react according to the decision. This brings us to the IoT paradigm and the definition of IoT, which is somewhat as follows.

The network of all of these smart devices that are connected to sense, communicate, and interact within them and with the external environment on real-time data and make decisions by processing these data is collectively called the Internet of things or the IoT. All IoT devices are connected centrally to the cloud to compute the enormous amount of data that IoT devices collect [10,11]. The cloud also has a vast data storage capacity and is generally remotely located from the devices. Furthermore, the cloud provides different kinds of services, such as software as a service (SaaS), platform as a service (PaaS), and infrastructure as a service (IaaS), heading towards anything as a service (XaaS).

The vast number of interconnected devices drives out huge amounts of data in a raw format that needs to be processed, and decision making needs to be performed based on that data in real time. However, only one cloud is remotely situated and serves many devices, all of which need to respond quickly to the data. Consider the case of a driverless car that sends all of its sensors’ data to the cloud to make decisions [12,13]. The car’s camera sends out the image of the signal turning red just now, but as the decision to stop comes from the cloud, it has already crossed the signal, causing an accident. There are many such use cases in the real world where decisions need to be made instantaneously. At that time, it was clear that there were better things to carry out than sending all of the data directly to the cloud and waiting for the response to act. Therefore, a new paradigm is known as edge computing or fog computing. Although most people attribute both as the same thing, and it is not easy to distinguish between them, there is a subtle difference separating them. Other important paradigms similar to these have also been developed, such as dew computing or mobile cloud computing or a hybrid of any of these, which will be discussed later in the paper [14]. Fog computing aims at becoming a mediator between IoT devices and the cloud by cloning activities performed by the cloud but with lesser strength than the cloud itself. This process aims at bringing efficiency and scalability in two ways: vertical (by reducing the time taken to reach the cloud) and horizontal (fewer data needed to process and manage) [15,16]. However, the cloud now has a role. The high latency problem of the cloud can be overcome by using idle resources of various devices near users. However, fog computing still relies on the cloud for complex processing, which does not require real-time attention. The fog itself addresses the tasks that need such real-time attention. Its abstracted view is passed on to the cloud for complex data analytics, maintaining a global view of the interconnected devices and pushing local updates to the connected fog nodes [17].

### 1.3. Need for Fog Computing in IoT

We have gone through the paradigms of IoT and the introduction of fog computing into the IoT paradigm. Therefore, now, we will dive deep into the discussion as to why we should be using fog or edge computing in the IoT-driven world and why it is so disruptive [18]. We will first look at how our network would look with fog computing.

Cloud has more power, while fog has more agility, and agility will beat power when real-time processing and decision making is involved. With great power comes great responsibility, so the cloud has to make computations for all of the devices. In contrast, the fog has to make computations related only to locally connected devices. The devices connected mainly consist of sensors or actuators, which generate huge amounts of data every second. These data are also very raw, contain noise, and need to be pre-processed before some meaning can be derived. For example, it has been shown that a typical driverless car generates several megabytes of data every minute. This is where fog computing might be useful. Fog computing extends the already existing concept known as edge Computing. The edge computing paradigm states that computing and storage are performed directly on the end devices or near them. At the same time, fog computing addresses near computing but is separated from edge devices. The fog computing model is a distributed computing or decentralized model as opposed to the centralized model of the cloud [19]. Every fog node forms a layer of the interconnected network with one another next to the layer of interconnected IoT devices. The role of these fog nodes is not only to process data generated by the devices but also to transmit the decisions to the cloud via other fog nodes so that all of the fog nodes can have a common view of the system as a whole [20].

## 2. Integration of Fog Computing and IoT

**Figure 2:**
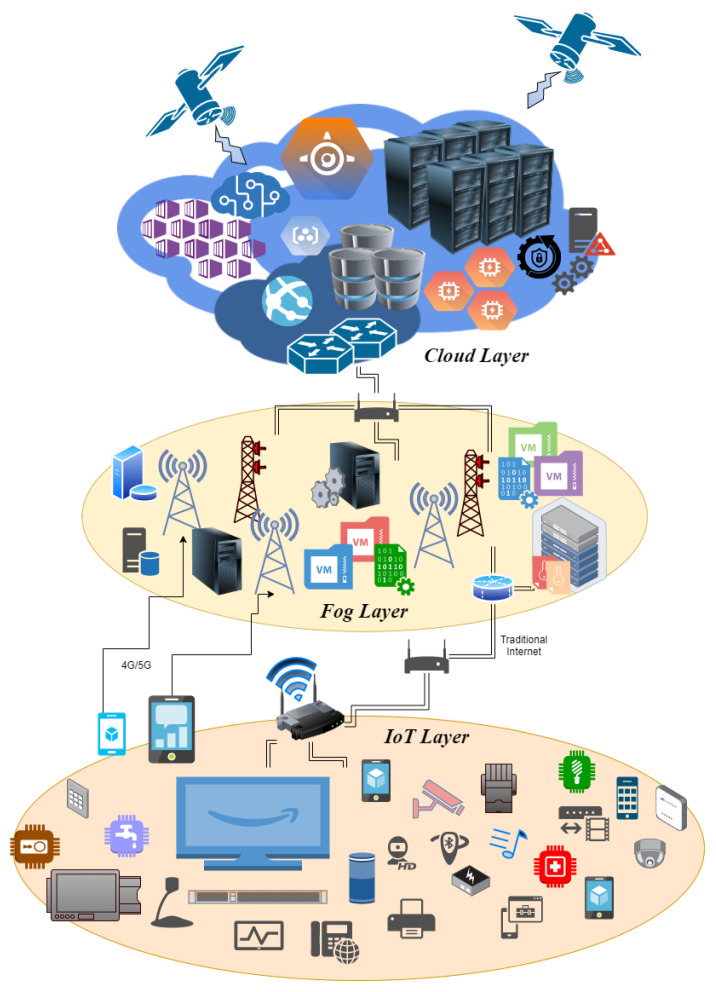
Layered architecture with fog computing.

The traditional cloud computing paradigm cannot resolve the low latency and faster response needs of time-critical applications, and that is where fog provides a solution to these problems. The fog computing paradigm effectively handles the problem of vast data collection from IoT devices with a low latency and faster response time, and support demands of quality of service [21]. Smart healthcare devices and wearable sensors monitoring patients will need an immediate response in critical situations, and fog can meet these requirements effectively. Fog’s architecture inherently supports agility and flexibility compared to the cloud, which may be packed with much power but does not provide agility, needing computation for sensor networks. Many works have proposed architecture with different perceptions that are most suitable for dealing with various problems specific to certain use cases. However, the fundamental architecture remains the same, with the three-tier architecture consisting of cloud, fog, and IoT layers. Figure 2 describes the architecture advocating programmability, flexibility, and efficient data analytics aspects of fog nodes.

### 2.1. Cloud Layer

The cloud layer comprises a cloud server loaded with huge data storage and processing capabilities and services provided as APIs or direct connections. This layer is responsible for collecting pre-processed data from connected fog nodes and storing them in persistent storage on the cloud. With ample computing and storage resources in the cloud, it will perform tasks that are not feasible in the fog servers, ensuring global coordination and control amongst all connected fog nodes and servers. It could also provide centralized services such as maintenance and security enforcement policies and ensure flexibility for accommodating future modifications. Inspired by modern trends in big data analytics and artificial intelligence, the cloud layer can be programmed to further improve the management and efficiency of fog layer components from centralized processing and to learn from data collected from the fog layer.

### 2.2. Fog Layer

**Figure 3:**
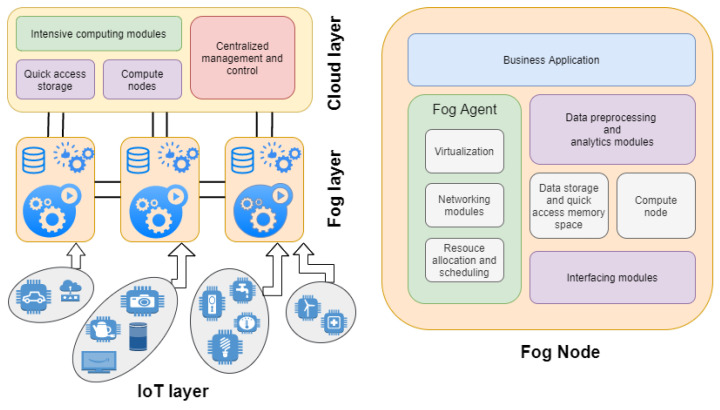
Architecture of fog node.

The fog layer resides between the cloud layer and the IoT layer and is a layer of heterogeneous nodes. For example, fog nodes could range from high-end servers, gateway devices, edge routers, computers, mobile devices, and smart vehicles to sensors with little processing capabilities or supporting different networking technologies, such as high-speed physical links or multiple wireless access technologies such as WiFi, 4G/5G, and LTE. For that matter, fog nodes have an abstraction layer that abstracts out the discrepancy of underlying hardware and technologies and exposes a uniform and seamless interface for management and control. Furthermore, multiple fog nodes interact with each other for data and processing coordination. Figure 3 shows the architecture of the typical fog node.

#### 2.2.1. Fog Agent

The fog agent handles the entire fog node management, which holds core functionality modules such as virtualization, network management, and resource allocation and scheduling. Physical resources are abstracted to the upper layers and provide support for creating virtual hardware components and environments for running processes and services in the node. Due to this mechanism, it is easy to allocate resources according to the processing needs at the run-time by creating virtual machine instances on top of the virtualized infrastructure with the help of HyperVisor. Virtual machines are created to host various services and applications to serve the IoT data processing needs with a dynamic allocation of required virtualized hardware. This functionality helps to efficiently allocate and scale processing needs to certain limits using resources in the node.

Fog has inherent challenges regarding networking, whether inter-VM networking or with an external device. For that matter, the agent has networking modules that elegantly handle complex networking tasks and provides an abstraction layer, simplifying it further. It uses VNFs to provide network services with benefits of hardware independence, a high resilience, quick replacement, and easy configuration and deployment. From recent advancements in networking, it can also be customized to use case-specific networking modes to improve the throughput and reduce the latency in data packet communication.

A fog node can deal with many simultaneous connections demanding various resources and services for different tasks. In that situation, the fog agent acts as a resource allocator and manager for serving virtualized resources and de-allocating when the task is finished. The agent includes service orchestration functionality and policies for life-cycle management with a global messaging bus to send control signals for synchronization. It also deals with secure inter-process communication and consistent data resource sharing between different VMs. Various resource allocation strategies are implemented in the resource management module. These should be selected carefully because allocating resources efficiently is necessary to reduce latency in communication.

In addition, the fog agent also provides APIs to satisfy programmability needs at a low level in the node architecture. This feature supports the customization of fog nodes to accommodate functionalities such as real-time data synchronization between multiple devices, defining QoS, creating custom computing policies, and centralized control with mobile devices.

#### 2.2.2. Interfacing Modules

Today’s easily accessible IoT devices pose a huge challenge of interfacing them with some uniform access method. Therefore, fog nodes must have interfacing modules through which data exchange between IoT devices is possible. They could be the standard interface or follow some proprietary protocol. Interfacing modules will handle all complexities, such as sense, the establishment of the communication channel, bandwidth, connection type, and the nature of the data stream regarding connected device specifications. One service loaded with these interfacing modules will be made available, continuously running in the background for handling incoming requests and servicing them in combination with other resources following the requirement of the connected device.

#### 2.2.3. Data Storage and Quick Access Memory Space with Compute Node

This component corresponds to the primary advantage of the fog computing paradigm by processing data on the network’s edge rather than the data going to the cloud. Fog nodes have sufficient storage and processing power to process the data from IoT devices. Devices working as fog nodes can have various types of memory and compute resources, so this flexibility needs to be addressed by the fog agent through virtualization. There could be many types of storage memory available at the fog node, such as local storage memory, and faster memory, such as RAM and cache memory. For the processing, the compute node will have processors and GPUs, and for machine learning and deep processing, the compute node will have TPUs and ML processors. Virtualized resources will be available for applications to serve incoming connections, and the fog agent will handle allocations on a requirement base.

#### 2.2.4. Data Pre-Processing and Analytics Modules

Data collected from IoT devices could be huge, so they need to be processed efficiently without wasting much processing power and time. Various optimized pre-processing and analytical modules are loaded in the memory to accommodate these facilities. They are designed to process data in a parallel manner, which is crucial for serving multiple devices requesting services. In addition, some fog nodes may have AI-enabled hardware for advanced prediction and learning mechanisms with corresponding libraries. For example, nodes with the Intel processor family “Myriad” have SDK with a neural network compiler and Google Edge TPU with TPU libraries in order to use dedicated resources for deep learning in fog computing [22].

#### 2.2.5. Business Application

This is an essential component dictating the behavior of the fog node. It is easily programmable on top of fog resources and defines the services provided by the fog. The application will determine the role of the fog node in the network with IoT devices, which could be simple interfacing and centralized monitoring, or much more complex, such as deep analytics on real-time data and synchronization in combination with constant checking with other services to provide complete IoT network management. Systems such as smart health devices systems, home automation solution, parking space suggestion systems, or smart city management could be developed with the help of a network of multiple fog nodes leveraging a distributed nature. Furthermore, the architecture of the applications could be inspired by a monolithic, micro-services-based model or distributed containerization to exploit various advantages of scalability and QoS [23,24]. For example, frameworks such as Fogernetes [25], based on Kubernetes, could be used to develop the distributed application.

### 2.3. IoT Layer

The IoT layer includes all IoT devices on the network. There could be two types of IoT devices: fixed and mobile. Fixed IoT devices are located in particular fields or locations, such as smart home assistants, smart door locks and ambient light sensors, RFID tags and sensors, air quality monitoring systems, and smart alarm devices connected with physical links or wirelessly. On the other hand, mobile IoT devices could be portable and easily carried by users such as smartphones, wearable IoT devices, and vehicles. These devices form a layer of IoT devices and are connected in an ad hoc fashion with the network, generally via wireless access. Generally, these devices have restrictions on the data that they can process, and a limited bandwidth. Therefore, their main function is to collect data and provide it to the upper layer for processing and storage. Different devices can have different interfaces to interact with each other, which poses a challenge in data communication between IoT devices for automation needs. However, interfacing modules in the fog agent solve this issue and provide a standard for easy data sharing amongst IoT devices with greater agility.

## 3. Data Analytics

### 3.1. Data Generation Sources

Due to the IoT revolution, almost everything is becoming a source for data generation. As a result, a tremendous amount of data are generated every second. These data need to be pre-processed before something useful can be derived from them because only some of the generated data are relevant or useful. This section will look at the various sources from which data get generated.

**Figure 4:**
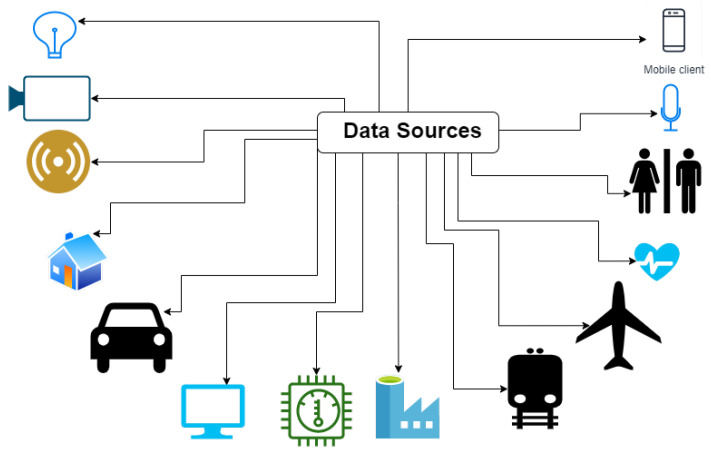
Data generation sources.

As we can see from Figure 4, typical data sources include mobiles, various types of sensors and actuators including thermostats, engines of airplanes, factories, mobiles, computers, automobiles such as driverless cars, metros, human health data, smart devices such as Google Home, Alexa Echo Dots, smart homes, smart shoes, watches, and, in general, all wearables, etc., and the number of items on the list increases all of the time. The data generated from these sources can be subtly classified into three major types of sources, which are as follows.

#### 3.1.1. Passive Data Sources

These data sources do not communicate data actively with the network or the intermediary nodes. These sources need to be pushed to the active state before data transmission takes place, and they transmit the data only when requested to them. These typically have a low power consumption and are present in remote places. We can take an example of a sensor that measures salinity in a sample. Here, the sensor will get active only when an API call is made to obtain readings.

#### 3.1.2. Active Data Sources

These data sources generate data continuously in the form of streams of data bytes, just like a driverless car. The data keep flowing to the nodes, as opposed to the passive sources, where we have to request data. Thus, the data need to be accepted in real-time, and the applications running on top need to be very accurate and sophisticated in the data transfer, as no data should be lost or misplaced. They should be handled well for further processing.

#### 3.1.3. Dynamic Data Sources

The data sources are termed as smart data sources. Here, communication with the application takes place instead of just data passing. The communication is bidirectional and also dynamic or in real-time. We can take an example of a surveillance camera with a small code of face detection embedded into it. When a criminal’s face is recognized, the camera can send an alarm message to the cloud-based criminal dataset program and communicate with it. Thus, these devices can not only send data but also alter the data format; for example, the camera does not send the whole image to the cloud, but only sends data in the form of whether the criminal has been identified.

### 3.2. Data Analytics: A Brief Introduction

**Figure 5:**
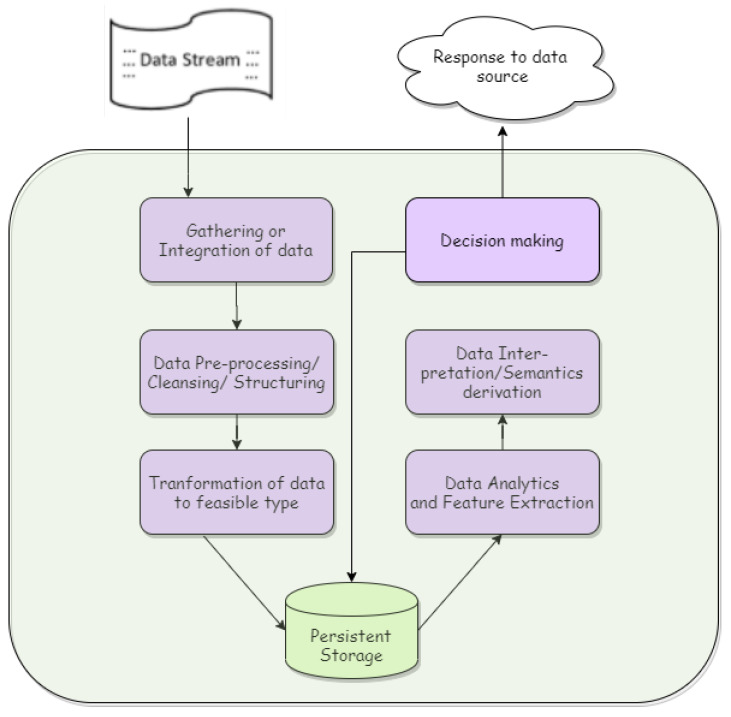
Flow diagram for data analysis.

Big data is a term that needs to be understood before we understand data analytics. It has been a topic of propensity to talk about and is just a byproduct of the Industry 4.0 revolution. It is a term that refers to the large amount of the raw dataset directly generated by the sensors or actuators. It can range from highly unstructured to highly organized and comes in various combined formats, including text, image, video, etc. To deal with such a huge amount of highly unstructured, complex, and hybrid data, we use data analytics or, more specifically, big data analytics. Big data analytics refers to a vigorous and convoluted analysis and deriving semantics to make accurate decisions [26]. Data analytics has become the most important task in the IoT and fog paradigm, as everything today is data-driven. The advent of big data also creates a new need in the market for data analysts and scientists, who require great skill and expertise to obtain some value from the vast amount of data [27]. The basic flow of data analytics is depicted by Figure 5.

### 3.3. Current Trends in Data Analytics

Data analytics has many opportunities and a large scope for improvisation considering its vast applications in the IoT and fog paradigm. Some of the current trends in data analytics include:

• Continuous intelligence;
• Graph analytics;
• Commercial AI/ML
• Conversational AI analytics/ NLP;
• Augmented data analytics;
• Automatic data and content management;
• Persistent memory servers.

We will look at a couple of them in detail below.

1. Augmented Data Analytics
  This is considered as the future of data analytics. The main motivation for a company to perform data analytics is insight generation from data. In the present scenario, the industry has a shortage of data scientists/analysts, and the need is increasing even more. The McKinsey Global Institute estimated that the U.S. economy could be short of around 250,000 data scientists by 2024. Even if the gap gets filled somehow, data scientists are not business experts; they can perform all tasks independently and must be under the constant scrutiny of business analysts. Thus, augmented data analytics is emerging to overcome all of the barriers because it reduces a company’s dependence on data scientists by automatically generating insights. It does so with the help of complex and advanced machine learning and artificial intelligence algorithms.
2. Persistent/In-memory storage
  Persistent storage is also one of the emerging trends, and will help foster data analytics even more. As the data generated are increasing exponentially, there is a current need for better ways to store these data so that they can be accessed rapidly with fewer latency issues. Persistent memory (PM) combines the byte-addressability of DRAMs and the non-volatility of disks and flashes. PM can be supported either through direct DAX or block access. The use of PM can be performed in three ways. Firstly, the applications can use it as an external or augmented storage entity and are not concerned about the non-volatility. In this case, the applications do not need any changes. Secondly, the applications can use its non-volatility property against DRAMs. Here, the applications themselves need to be modified to use the persistence property of PM. In the third case, the applications may use just PM instead of flash or drives.

### 3.4. Role of Fog Computing in Data Analytics

**Figure 6:**
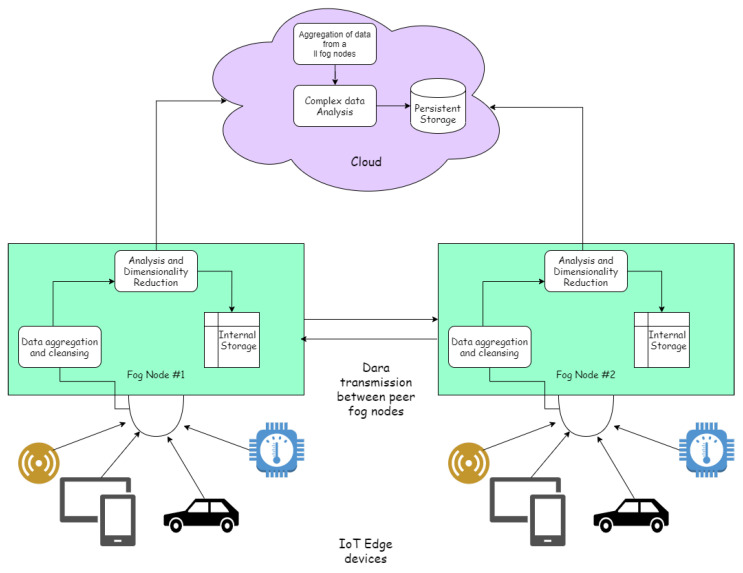
Data analytics using fog computing.

As we have already discussed, big data contain the following characteristics: volume, velocity, variety, and veracity. Dealing with all of these for real-time applications by ensuring availability and deliverability is a cumbersome task for the cloud. Data processing and analytics need to rely on robust and highly scalable messaging systems, commanding software engines for data stream processing and scalable data storage solutions. Fog computing has much potential for solving issues with huge data storage and for quick data analysis to respond to numerous events that call for prompt decision making and action [28]. Therefore, we introduced the paradigm of fog computing earlier in the paper. Now, we will see how fog computing will help us to achieve all of the benefits that it is known for, especially in data analytics. The role of fog computing in data analysis and the segregation of jobs from the cloud is best explained by Figure 6.

Fog computing presents a distributed method of computing, as opposed to the holistic approaches of the cloud [29]. Fog data analytics is a field where fog computing is used for data analytics at the edge of the network to reduce latency for applications needing a high availability and faster response times. The fog nodes are installed near the edge of the network, and they distribute the load of data going to the cloud by using virtualization techniques and absorbing all of the raw data from the IoT edge. A fog node can be thought of as a mini cloudlet. Then, these nodes can also perform data analytics on their own, responding directly to the devices, offloading local insights to the cloud in the background, and communicating with neighbor nodes about the learning. Augmented data analytics is widely used for automatic data processing and insight generation. The fog nodes cannot perform all data analytics work like the cloud due to the little resources of the cloud, but they are sufficient for local analytics. The cloud then performs a more complex analysis of all of the data collected from different fog nodes for obtaining global and heuristic insights into data [30].

## 4. Current State-of-the-Art

In the era of faster communication and even faster computational needs, fog computing prevails in the industry with such requirements by processing data closer to the source. Modern advancements such as big data analytics, machine learning, and deep learning are combined with fog for generating data analytics on the fly with adaptive learning [31,32,33,34]. Leveraging the technology of blockchain to accommodate privacy and security features in the fog layer is proposed in [35,36,37]. Software-defined networks (SDNs) with customized routing protocols are used to increase the energy efficiency and performance between fog nodes [38]. With SDNs and named data networking (NDN) [39], traditional networking concepts are redesigned to harness new advantages in fog computing. They drive innovations in various fields and transform the experience for greater efficiency and convenience. Recent developments such as quantum computing paradigms are also considered to optimize the performance of fog-based systems for parallel processing and dynamic load scheduling using quantum-computing-inspired optimization (QCIO) [40]. Finally, some areas harnessing the power of fog computing with IoT devices are explored for state-of-the-art implementation.

### 4.1. Smart Factories

IoT has enabled automation and obviated the need for human interaction, adding new capabilities, such as logging every event and making intelligent decisions at certain times. Having such advantages, IoT is most suitable for driving automation at various levels in the industry. For IoT technology used in the industry, there exists another specific term: the industrial Internet of things (IIoT). These IIoT devices include actuators, RFID tags, robots, flow control and conditioning devices, and sensors for various manufacturing, testing, and quality control applications. The cloud with these devices provides centralized control and helps to automate tasks with deploying applications on the cloud, managing IIoT devices communication and data analytics [41]. However, this implementation needs to deliver the expected value for some time-critical systems because of latency issues due to the round-trip distance between IIoT devices and cloud services. Fortunately, the problem can be remedied by enabling fog computing with IIoT to handle real-time data processing and analytics. Fog nodes host monitoring and control services that make it possible to serve low-latency requirements as they are closer to the IIoT devices, along with the implementation of SDNs to develop control plane behavior and employing time-sensitive network (TSN) protocols for optimizing and sustaining the network’s effectiveness [42]. With fog nodes with a sufficient processing power, real-time data analytics, such as the prediction of quality and estimation of faults, are generated with a high availability.

### 4.2. Healthcare

The healthcare industry is going through a total paradigm shift from cloud-based applications to fog-based IoT device data management. This transition is due to its requirement of a high availability with geospatial awareness and an instant response to events reported by health-monitoring sensors. Body area networks (BANs) are most popular for monitoring the health of individuals without any human observer in a friendly way. They also provide notifications to relatives and instantly contact emergency services with complete automation. Fog computing power requirements of healthcare have severe time constraints. Hospitals deploy fog nodes for collecting data from nearby IoT devices and accommodate the need to monitor the health condition of many patients. Fog enables smart healthcare, which notifies appropriate staff members when patients are in critical condition with their geospatial location. Various methods have used deep learning to detect epileptic seizures [43], heart disease [44], and cancer classification [45] in combination with fog nodes and data from IoT devices on the fly. Systems such as fog ambient assisted living (FAAL) [46] have been developed to help people suffering from neurological disorders, and personalized healthcare support for remote patients with diabetes is making a tremendous impact on people’s lives [47].

### 4.3. Home Automation

IoT devices have a huge role in home automation, making it a smart home with connected devices and data synchronization over the network of IoT devices. These devices are connected with wireless access technologies and can make intelligent decisions according to situations and conditions. Devices such as intelligent lights that adjust the lights according to the available sunlight at a given time, smart thermostats that dynamically adjust temperatures in combination with devices such as air conditioners or heaters, and security cameras that intelligently recognize people and track their movements are driving home automation and the quality of living to new heights. Data from all of these devices need to be processed in real-time for the best experience and to receive immediate support in emergencies. In addition, with adaptive learning mechanisms to constantly shape the services of all of these smart devices to serve the best experience to the user, fog nodes embedded with machine learning and deep learning hardware provide fully customized and user-centric automation without any direct human interaction. In modern scenarios, all smart IoT devices are connected to the central network and can be accessed and controlled from mobile and wearable devices. These smart devices, in coordination with each other, through sharing real-time data, have also resulted in an overall reduction in energy consumption.

### 4.4. Vehicular Networks

The number of vehicles on the road significantly increases each year. Managing communication more securely and effectively is more difficult due to urbanization and population convergence. A driver mistake is a major factor in many accidents and catastrophes. Because of this, it is crucial to monitor driver behavior [48] continuously. The concept of connected things is popular; it has made its way to the network of vehicles to exchange data using wireless technology and several other devices, such as RSUs and computational resources, present in the vehicles. Data aggregation and communication become even more complicated when multiple sensors, in addition to sensors for the vehicle and surroundings, perform this function. Intuitively, these networks are highly latency-sensitive and need a quick response, which could be accommodated by using fog computing and computational resources present in the vehicles to act as fog nodes. This implementation supports a low latency in data communication between RSUs and vehicles, and vehicle-to-vehicle. communication happens by sending different types of messages to nearby vehicles. These messages could be a query, response, or cooperative, serving the purpose of intelligent data sharing [49]. The vehicular network has great advantages as it helps the driver to safely change the lane, provides estimated congestion at certain points, and suggests alternative routes to avoid congestion. The smart network of connected vehicles implemented with the SDN approach further amplifies its capabilities and advantages [50]. Many traffic signals combined with vehicular networks would result in effective traffic management and a reduced waiting time [51].

### 4.5. Disaster Management

Currently, many cameras and monitoring devices are available throughout the cities, which can detect disasters such as fire, smoke, or crashes. Processing live streaming data from the sources as a method of identification could be a task that needs immediate analytics generation and decision making. Conventional cloud-based solutions would be scalable but suffer from a lack of processing on edge devices to make quick decisions, resulting in a higher latency. The implementation of fog makes edge computing possible, satisfying real-time analytical needs for taking certain actions. Systems using fog computing to forecast and monitor disastrous phenomena [52] at coastal areas and ensure the safety of the people and infrastructure have also been proposed. The same could be implemented in the smart home environment for fire and smoke detection [53] at any location with the help of sensing devices and fog nodes. Disastrous events such as a gas leakage, fire, and short-circuit could be averted with fog processing and IoT sensing.

## 5. Research Challenges

### 5.1. Low-Latency Transmission

IoT devices generate a large amount of data, so we need efficient data processing capabilities to handle that large amount of data within certain time constraints. On the other hand, real-time latency-sensitive applications pose challenges that are not solved only by efficient data processing but also stipulate that the processed data are within a few milliseconds. Some solutions have been proposed to satisfy these requirements, but active research remains because of the inherent complexity and design challenges.

The challenge of low latency is amplified dramatically when many devices depend on each other for data and responses. Data must often be processed to some extent so that they can be served to another device because of that particular device’s interfacing and structural data needs. At this point, if the latency constraint is not taken into account correctly, it will hugely impact the performance and effectiveness of the system of inter-dependent IoT devices because there is already a delay due to some level of pre-processing. A higher latency will worsen the situation, resulting in a waiting time for data and a poor performance, eliminating the comparative advantage of fog computing. Table 1 compares various works that show latency sensitiveness in different areas.

As a partial solution, Dew Computing can be considered by packing pre-processing capabilities within the devices and establishing a direct communication path between them. However, if devices could use data from multiple devices, this approach could be more economical, and another alternative should be explored. For example, Magurawalage et al. [54] suggest the concept of aqua computing for improving the user experience and offer clone architecture to accommodate lower latency requirements by cloning user-specific data in the edge devices so that they are directly accessible to the user without any delay. The Internet provider is considered to manage these clones to serve the user on demand and is responsible for updating them for the time being. However, managing user-specific clones would be costly in terms of resources.

Additionally, no perfect universal solution can exist for low-latency applications, and some trade-off is needed for the high availability, latency, and redundancy. If we consider an application-specific solution, then a large amount of heterogeneity would be added to the network, resulting in difficult tasks to manage.

**Table 1:** Research work on latency sensitivity in different areas.

Work	Scope	Focused Aspects	Strength	Weakness
Naranjo et al. [55]	Smart city	Latency, energy consumption	Lower energy consumption, heterogeneous communications between IoT devices	Low scalability, lack of real-time data processing
Singh et al. [56]	Smart grid	Network utilization, latency, energy utilization	Context-aware information with reduced latency, better energy and network usage	Lack of resource cost measures, high computational complexity, unevaluated overhead
Mahmud et al. [57]	e-Healthcare	Latency, energy consumption, network utilization	Lowered energy consumption with low response time	Mobility is ignored, latency caused by high computation
Chamola et al. [42]	Fog implementation with cloudlets	Response time	reduced network latency for SDN	Energy consumption was not evaluated, latency caused by high computation
Romeo et al. [7]	Robotics application	Power consumption, latency	Modeled battery discharge profiles, reduction in power consumption, low latency	Accuracy was not investigated, low scalability
Alam et al. [58]	Mobile application	Response time, energy consumption	Low execution time and low latency, suited for multi-agent architecture	Mobility, privacy, and context awareness were lacking
Ahn et al. [59]	General	Energy, wait time	Considered gap in the wait time, energy expenditure for different devices, latency	Heterogeneity was not considered, inter-dependency of IoT devices, high computational complexity

### 5.2. Heterogeneity and Interoperability

Myriad IoT devices [60] present in the market have a different set of data and interfacing requirements, which poses a great challenge in integrating it with an entire network without incurring the additional cost of computation and hardware. Devices such as thermostats, ambient light sensors, fire and smoke detection sensors, digital voice assistants, smart door locks, and connected media devices induce a large amount of heterogeneity in the network, and data communication between these devices needs a uniform interface. Fog nodes solve interfacing issues by abstracting the interfacing complexity and providing a uniform standardized interface for easy communication and real-time data stream sharing. Furthermore, fog node communication protocols should be designed in a way that can cope with future changes. Some protocols proposed in [55,61] are used for energy-efficient routing and navigation in heterogeneous networks.

**Figure 7:**
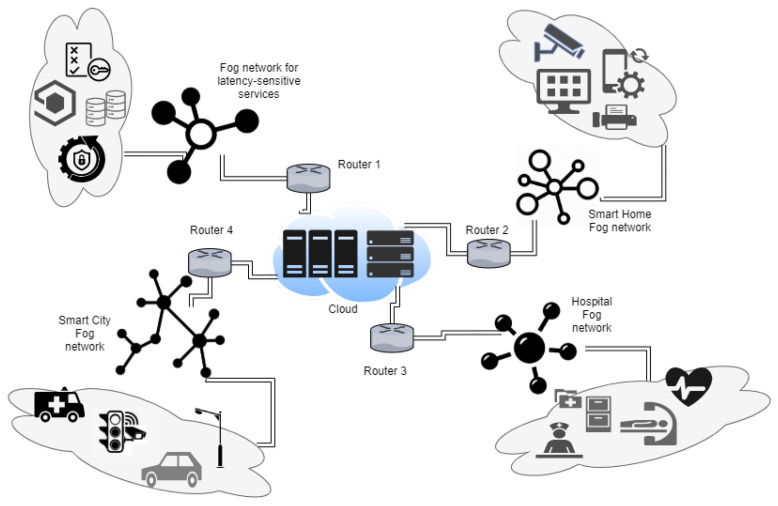
Fog network of heterogeneous devices.

The fog layer could be deployed as distributed fog nodes with someone acting as the controller node for larger system requirements. Here, too, nodes need to be of different types, and they still should work in harmony. In case of failure of the controller node, some other node should act as a controller to handle the operations without any problems and continue to serve connected devices. Devices should be easily connected to the network, and data transmission between different types of devices should be seamless to support the best user experience. In smart homes, most devices are connected to a central wireless network and can be easily operated and controlled with different mobile devices. For example, digital assistance and a stereo system connected with the wireless network are controlled using smartphones or tablets, which should support platform independence and uniform APIs. The heterogeneity of devices can be observed from Figure 7.

### 5.3. Programmability

Applications deployed on top of the fog nodes are meant to serve the user’s specific interests. The type of data storage and nature of pre-processing and data sharing could differ from one use case to another. Therefore, fog nodes should accommodate programmability needs without restricting the platform and underlying hardware. For example, a computer operating on a Windows operating system could act as a fog node or a smartphone with Android; different characteristics and capabilities of devices are taken into account when deploying an application. A smartphone cannot handle continuous data processing tasks because of battery constraints; therefore, the programmability of these types of devices is limited, and similar considerations should be figured out when designing a fog computing environment. It may be challenging to develop a generic fog node setup that is compatible with many devices.

If the fog node is equipped with high-end processing resources, it could be leveraged to improve data analytics and storage capabilities, which would help to adjust fog network parameters to maximize the quality of the user experience. Machine learning and deep learning can be employed for that purpose. Many works have proposed such solutions in [62,63,64], helping to reduce the energy consumption [56], low-latency delivery, and QoS, as well as bandwidth optimization. However, the complexity of the technology stack and architecture of the fog node will directly affect the effectiveness of the data analytics services deployed on the fog node. Fog nodes have a limited data storage capacity compared to the cloud, so data that could be cached into the fog node for quick access are limited. Therefore, appropriately policy managing the cache in such a way that minimum external contact is needed and no unnecessary data remain present is necessary. The author in [65] proposes a new caching scheme known as the Steiner tree-based optimal resource caching scheme. Fog nodes use this tree to reduce total path costs and minimize resource caching costs. However, if the fog layer is composed of many other fog nodes, it further complicates the policy that provides a high availability, consistency, and coherency.

### 5.4. Quality of Service

The sheer amount of requests and network traffic from connected devices jeopardize the network performance. This results in significant delays, broken data streams, dropped calls, and glitches in video calls. Moreover, only some of the services in the network hold equal priority; some time-sensitive application [66] needs to be serviced quickly and continuously with required resources; otherwise, it would result in troublesome delays and fragmented data packets. On the other hand, applications such as a file transfer could bear some delay in the data transfer and still have intact packets. Therefore, ensuring the QoS is essential for managing the network effectively and delivering the values demanded by different services.

The need for QoS parameters such as reliability, the continuous availability of bandwidth and low-latency servicing, resource provisioning, high-priority data transfer, and mobility poses a great system design challenge. Real-time data application needs a continuous bandwidth and low-latency servicing for the best user experience. A revolutionary black-box multi-algorithm described in [67] shows how end-to-end latency might be reduced by 60%–70% while largely utilizing the temporal locality. The processing and networking time are the two parameters used to measure the end-to-end latency. Similarly, the fog node must be able to prioritize data processing needs and serve required resources so that the execution does not introduce any further delay. Authors in [68] proposed a method for estimating resource allocation, based on how many resources will be utilized according to historical data and customer usage patterns, named media fog resource estimation (MeFoRE). Table 2 compares various works by authors in their research and shows which QoS aspects were considered by them.

With traditional networking, fog computing is refrained from its true potential for QoS and flexibility with a lower latency by constantly learning solutions from modern developments. SDN gracefully decouples the control plane from the data plane and emulates centralized control over the data plane. A fog layer implementing SDN networking would benefit from flexibility and data forwarding control offered by SDN. A fog node can work as an SDN controller that guides the data plane according to network requirements, which will ultimately help to improve the QoS and efficiency of data analytics tasks, eliminating latency issues.

Fog-based systems can deliver a high-quality user experience to end users. First, the preference of services is decided based on the behavioral usage patterns, geographical location, and mobility context, and then the user experience is optimized from gained values of parameters. The work of [69] describes delivering the QoS to users in IoT ecosystems that leverage a virtual infrastructure proposed as the self-organizing fog of things (SOFT-IoT). The fog of things gateway has been set up to work as a smart device based on fog computing in the environment.

### 5.5. Scalability

Scalability is among the main characteristics, the need of which gave birth to cloud capabilities near the end devices so that, instead of overwhelming the cloud with a huge amount of data, fog focuses on filtering it at the fog layer. By 2022, USD 2.5 million will be spent every minute on the IoT, and one million new IoT devices will be sold every hour. Now, processing at the edge would help to overcome the problem more efficiently at this scale. However, not only computing at the edge but also the coordination of such a large number of devices needs to be handled as requirements grow over time. This presents the challenge of designing a scalable solution to accommodate the data analytics needs of collected data from connected devices.

A variety of use cases have different approaches for solving the particular problem, and they might be inherently disparate, serving those specific requirements. For example, for the management of smart home devices, one fog node equipped with sufficient resources might be enough. However, regarding connected devices throughout the city, a large number of fog nodes is certainly required. In addition, the resources need to be scaled with the increased number of users. The relevance of placing services at various levels of the tree topology, which is rooted at the cloud data center, is discussed in [59] to optimize the performance and scalability. The work [70] presented multi-tier fog architecture consisting of ad hoc and dedicated nodes with dedicated and opportunistic computing resources, primarily focusing on maximizing analytics service utilities. The authors in [71,72] proposed systems that support real-time efficient data processing for vehicular fog networks and easy traffic management.

**Table 2:** Comparison of QoS aspects from various works.

Work	Scope	Quality of Experience	Energy Efficiency	Delay Sensitiveness	Reliability	In-Network Caching
Stojme vic et al. [68]	Machine-to-machine networks	✓		✓		
Huang et al. [66]	Vehicular networks	✓		✓		
Craciunescu et al. [73]	e-health applications	✓		✓		
Dantu et al. [74]	Smartphone-based applications	✓		✓		
Sarkar et al. [75]	loT-based applications		✓	✓		
Dastjerdi et al. [76]	IoT-based applications	✓	✓	✓		
Zhu et al. [77]	Website rendering			✓		✓
Hu et al. [78]	Mobile applications		✓	✓		
Hao et al. [79]	Ubiquitous computing		✓	✓		✓
Mubeen et al. [80]	Automation applications			✓		
Shih et al. [81]	Radio access networks		✓	✓	✓	
Prazeres et al. [69]	loT-based applications		✓	✓	✓	
Flores et al. [82]	Social-aware device-to- device communication			✓	✓	
Fan et al. [83]	Web-based applications			✓		✓
Wang et al. [54]	Device-to-device communications		✓	✓	✓	✓
Su et al. [65]	Latency-sensitive applications		✓	✓		✓

Not only the placement and efficiency of fog nodes but also the number of fog nodes and type are the factors affecting the network’s performance. It is also a necessity for the placement of nodes to be flexible according to the demand at a certain time. In the case of a smart city, fog placement should be dynamic enough to support changing the number of users at any time and to continue operating and servicing users. To support this goal, ref. [84] leverages the mobility characteristic of buses to deploy fog nodes, presenting a fuzzy-based real-time auto-scaling (FRAS) mechanism. Auto-scaling will surely help to improve the overall performance and QoS, even at times when there is a high load on resources. The author of [85] integrated the hypervisor technique with container virtualization and constructed an integrated virtualization fog platform for deploying industrial applications based on a virtual network function. The FRAS mechanism presented in the paper provides a dynamic, rapid, lightweight, and low-cost solution to service auto-scaling problems.

### 5.6. Authentication and Access

As we have mentioned earlier, IoT devices are increasing exponentially. These devices access and share resources/services such as storage, PaaS, and SaaS with the omnipotent cloud [86]. Thus, the authentication of these devices becomes the responsibility of the cloud in order to identify legitimate users and keep any other person or bot with malicious intent at bay. Sometimes, these illicit users attack the servers and keep their servers busy by bombarding spam requests. IoT devices are quite vulnerable to cyber-attacks, especially Dos and DDos attacks [87]. Thus, a robust authentication technique with a quick response time must be used. Some people claim that using a cloud-based technique prevents the devices from malicious attacks [88]. Although authentication via the cloud is secure and robust, it has a latency problem and is also not scalable [89]. Hence, we have to accept the help of fog nodes by performing some authentication processes at the edge in fog nodes [30].

Authentication and access for the end devices is a challenge in the fog architecture due to many reasons. Firstly, the fog architecture already comes with a resource constraint as it has to serve many devices with limited resources. Thus, ensuring the continuous availability of fog nodes is also a challenge in the authentication scheme. Secondly, if fog nodes have to carry out authentication at the edge, they either have to perform the authentication or communicate with the authenticating agent through API calls. This task increases the workload of the data transfer channels of the fog node, and the available bandwidth also reduces security-related threats because of the transfer of sensitive data, such as credentials near the edge. Therefore, many authentication schemes have been proposed to date. One of the techniques used is the single sign-on (SSO) authentication scheme. In SSO, a single login is required, and it provides access to all of the services within the system, where no further authentication is necessary, but this scheme has a backdoor for man-in-middle attacks [90,91,92]. Moreover, if the SSO provider gets compromised, then the security of all other devices under that are compromised as well [86]. Therefore, multi-tier and multi-factor authentication schemes are used to ensure that the security of users is not compromised.

From the above discussion, it is clear that authentication and access is a double-edged sword. If more focus is placed on making the authentication system more robust by introducing a multi-tier authentication architecture, user experience and convenience is at risk. On the other hand, if user convenience is catered for, the security might get compromised. Dealing with both simultaneously by maintaining the right amount of balance between the two is one of the greatest challenges in authentication in fog-enabled architecture. Table 3 summarizes the various authentication techniques implemented to date, along with their pros and cons.

### 5.7. Prediction and Optimization

One of the major applications of fog architecture is vehicular networks because of their low latency requirements and continuous and uninterrupted availability. Much is at stake regarding handling vehicular networks through the fog environment. The main intricacy here is that the vehicles are constantly moving at different speeds [101]. On the contrary, the fog nodes that they connect to do not move and are stationary or anchored at specific points on the streets. Thus, during motion, the vehicles need to constantly connect and disconnect to different fog nodes that come their way very quickly so that they can perform the actual communication that they intend to. This is where the role of prediction becomes important. Latest and state-of-the-art machine learning and deep learning algorithms are used for a better prediction accuracy. Some authors have also emphasized using caching policies [102,103] for content popularity and user preference prediction using the online gradient descent (OGD) method [104], prediction in terms of choosing the nearest fog node, and also cost prediction. The challenge here also lies in the state transfer during the constant connection and disconnection with the fog nodes. As the vehicles move, they detach from present fog nodes and reconnect to the next. Therefore, the vehicle’s state needs to be transferred to the next fog node to perform further computations and reduce the overhead of calculating everything repeatedly.

Optimization is the biggest challenge in fog computing at present. Optimization, both in terms of availability and cost, is necessary to ensure on-demand serviceability, efficient resource allocation, energy consumption, security, reliability, resource usage, and bandwidth optimization, as well as thwarting unnecessary overheads. Optimization can also be talked of in terms of data-driven, code-driven, task-driven, or a combination of these. Many parameters can be considered for optimization in the fog-driven environment, as the performance depends on many parameters. An optimization problem can be defined according to the application by setting constraints on some parameters to achieve desired results [105]. For example, the power consumption constraint applies to end devices because they run on batteries, whereas this is not the case with fog nodes as they do not operate on batteries. The challenge here is that, although individual optimization problems have been defined, they are yet to be integrated into one coherent and cohesive unit for overall optimization. Thus, we need to find different optimization techniques for different sub-optimizations within the system. As we already know, the fog environment is a distributed environment, so any algorithm applied for any purpose in the fog should be distributed. This is also a challenge in optimization, as designing a distributed algorithm requires special skills and knowledge of the distributed architecture domain.

In big and convoluted networks, there is generally a multi-tier fog architecture with more than one layer of fog nodes. The computation power of a fog node is directly proportional to its distance from the edge, which means that the farther a fog node is from the edge, the more computation power it has. Still, because of being far away, it is associated with a latency overhead. This imbalance is termed the power–latency tradeoff. Therefore, when the workload on a particular fog node increases, it can offload part of its workload to the next layer’s nodes, and this is where optimization is needed. The authors in [106] mentioned a novel approach for predictive offloading and stochastic network optimization in resource allocation by using a queuing model for optimization.

### 5.8. Orchestration

The fog paradigm has envisioned a distributed computing and allocation approach as opposed to the centralized approach of the cloud paradigm. Different orchestration techniques have been implemented in the cloud. Orchestration can be defined as a sole, uniform, and centralized approach that accounts for coordinating communications among different applications and services. Thus, orchestration is related to automating the interaction processes among various services, but applies to centralized systems. However, as we have seen, fog uses a decentralized approach with benefits. Moreover, fog has some other characteristics apart from its decentralized approach, such as resource constraints and heterogeneity. Thus, introducing orchestration into the fog paradigm is a challenge. In this context, a novel concept called choreography can be employed. Choreography refers to maintaining a global view of all applications or services across the edge devices through information sharing, thereby updating all local changes to the global view. Thus, choreography uses a distributed approach, performs orchestration-related functionalities, and could be our solution for implementing at the fog–edge interface/link or the southbound region.

Many different orchestration agents have been proposed by different authors with their detailed architecture, which is summed up in the paper by Karima Velasquez et al. [107]. The concept of virtualization has been used by many authors for the same purpose [108], while some authors have also proposed containerization in orchestration in the fog environment [109]. Most authors also propose software-defined networking (SDN) for obtaining centralized control of the network and an automatic management of the global view of the network as soon as any local changes occur. However, Jaeger [110] proposed an orchestration architecture based on network function virtualization (NFV). The general approach by many of the authors has been focused on sub-problems, and very few authors tend to choose a hybrid approach involving both orchestration and choreography, with orchestration at the north-bound region (between the cloud and the fog) and choreography at the southbound region (between the edge devices and the fog). Table 4 shows some related work in the field of orchestration.

### 5.9. Resource Scheduling and Allocation

Cloud computing can be thought of as an infinite warehouse of resources. Still, latency is always an issue due to the plethora of devices connected to the cloud, limited bandwidth available, and geographical separation of the cloud from devices. To tackle this, we introduced the concept of fog computing. However, due to the geographical vicinity of the edge devices, fog nodes are generally less powerful than the cloud. Moreover, they contain limited resources compared to the cloud. This makes achieving maximum throughput through scheduling and resource allocation challenging. When the term resource management is discussed, it is an amalgamation of load balancing, task offloading, resource provisioning, and allocation. Here, we discuss resource scheduling and resource allocation.

Resource scheduling can be thought of as the optimization of the assignment of various tasks submitted by the edge devices to the fog by meeting the required QoS levels while also ensuring time complexity. Thus, resource scheduling is an optimization solution for scheduling a set of submitted tasks T1, T2, T3, …, Tk, to a set of fog nodes F1, F2, F3, …, Fp, with various QoS requirements, such as cost, time minimization, or the availability and optimization of the optimization function for scheduling time [115]. The resource-scheduling problem is considered an NP-hard problem and uses meta-heuristic algorithms to find feasible and near-optimal solutions in linear time. The resource scheduling approach is divided into three categories based on the time when the scheduling takes place. Static scheduling takes place when tasks simultaneously reach the fog nodes, and the decisions for scheduling are made before submitting tasks [116,117,118,119]. Thus, there should be prior knowledge of the demands and available resources for this type of schedule to take place, which is not always the case, as obtaining all knowledge beforehand is not possible in all cases in the fog environment. However, the dynamic scheduling algorithms [76,83,120,121,122,123,124] do not require all prior knowledge, and the scheduling of the tasks takes place after the tasks get submitted to the fog nodes, thus allowing for more flexibility for scheduling algorithms. Some authors [57] used a hybrid approach involving both static and dynamic scheduling according to the use case. Table 5 recapitulates all different scheduling techniques.

Resource allocation is considered to be an important factor in resource management. Resource allocation in the cloud is different than that in the fog. This is because the cloud is a single cohesive unit with infinite resources, so resource allocation is not a big issue in a cloud environment. Still, the fog nodes are geographically widely distributed and there are limited resources available. As a result, resource allocation in the fog environment is difficult [128] because, in addition to the fog’s distribution, there are also QoS requirements for IoT devices that must be met. The approaches to resource allocation can be broadly classified into two categories: auction-based and optimization-based. The auction-based approach [129,130,131,132] is similar to a real-time auction, where the IoT devices bid for the available fog nodes and the fog node is sold (allocated) to the highest bidder. A specified auction mechanism is employed to allocate the fog nodes to the IoT resources. The fog nodes listed for bidding are according to the required QoS and other constraints that specific IoT devices require. In the optimization-based technique [58,133,134,135,136,137,138,139,140], a double-matching problem is formulated wherein cloud devices and the fog nodes are coupled for the IoT devices. The problem is considered to be an NP-hard problem for finding an optimal set of fog–cloud pairs for IoT devices while fulfilling various QoS requirements.

## 6. Case Study: Fog Data Analytics in Healthcare

Advancements in technology have significantly benefited the area of healthcare. Recent developments in IoT and healthcare have improved patient healthcare services. Healthcare services are critical; we need instant solutions to problems and real-time patient services. Smart healthcare devices are increasing significantly and generate huge amounts of data that need to be processed and analyzed in real time. Fog computing plays a vital role in managing large volumes of health data and has benefits of a reduced latency, improved energy efficiency, increased reliability, and improved energy efficiency. This case study focused on the health monitoring system using fog computing. Rather than sending the big data generated by the health devices to the cloud layer, they will be processed and analyzed at the fog layer through fog nodes (FNs) to make real-time decisions. Fog computing plays a vital role in healthcare applications: they can decrease the network’s data flow and provide an improved latency, security, and preventive care to patients [141].

**Figure 8:**
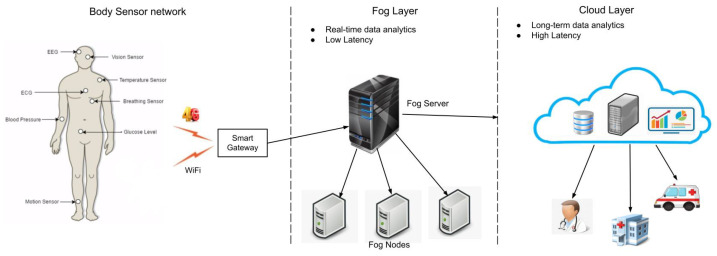
Fog data analytics in healthcare.

In this case study, a fog-based healthcare scenario was implemented, where patient health data were collected and transferred to the fog nodes at the fog layer. These data were filtered, pre-processed, and analyzed, and real-time decisions were made for the better treatment of the patients. Figure 8 represents the IoT-based fog-enabled model for healthcare. Patient data were collected from ECG sensors attached to the patient’s body and sent to a smart gateway. Based on the criticality level of the data, the captured data were sent to either the cloud or the fog layer by the smart gateway. The data received at the fog layer will be processed and analyzed to provide timely medical treatment to the patients, reducing latency. If the gathered data are not urgent, they will be sent to the cloud for further analysis and long-term storage. The patient’s health information is accessible remotely by family members and medical personnel from both the fog and cloud layers.

The performance of the proposed fog computing model was analyzed through a simulation and experiments using the iFogSim simulator. The simulation measures latency and network utilization in cloud and fog computing environments. The physical topology in iFogSim includes various ECG sensors, fog devices, and cloud servers. The simulation was conducted with five different physical topology configurations—conf1, conf2, conf3, conf4, and conf5—with monitoring devices 4, 8, 16, 32, and 64, respectively, and measured the latency and network utilization. The monitoring devices used in the configurations had a CPU length of 1200 million instructions and a network length of 20,000 bytes. Table 6 and Table 7 present the simulation results of latency and network utilization, respectively, for each configuration in the fog and cloud environment.

**Figure 9:**
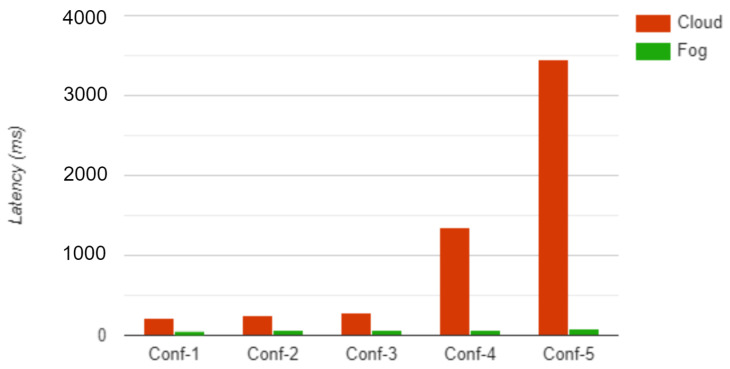
Latency.

**Figure 10:**
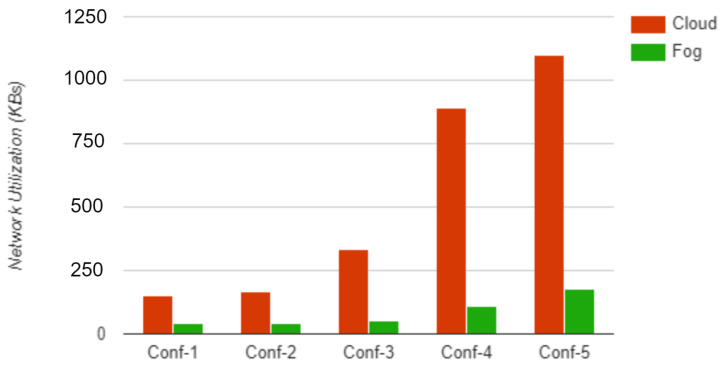
Network utilization.

Figure 9 and Figure 10 show a graphical comparison of the results for the different configurations. The simulation results show that using fog computing will improve the latency and the network utilization. Fog computing improves the quality of service of the complete healthcare system.

**Figure 11:**
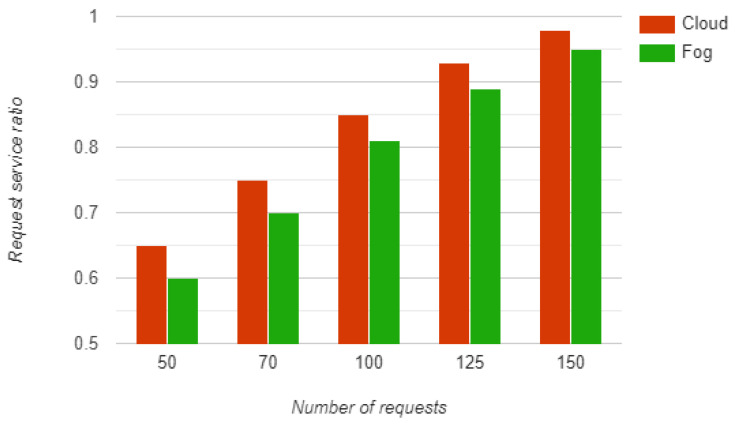
Request service ratio.

Fog devices have limited resources compared to the cloud. We evaluated the request service ratio in both the fog and cloud environment. The simulation results show that the cloud environment has the better request service ratio compared to the fog environment due to the limited computational capability of fog devices. Figure 11 shows a graphical comparison of the request service ratio in the fog and cloud environment for a different number of requests.

## 7. Conclusions

The exponential growth in sensors and smart devices produces significant heterogeneous data. To manage such data, we need efficient solutions deployed near the devices. Fog computing, which works as a middle layer between the cloud layer and the IoT devices, is a solution to the problem of real-time data delivery, especially in critical applications such as e-healthcare systems. Fog data analytics is an emerging solution for handling the huge amount of data produced by smart IoT devices. This paper gave an overview of fog computing, the need for fog computing in IoT, data analytics, and the need for fog data analytics in IoT. The current state-of-the-art in fog data analytics in IoT with various use cases was also covered in this paper. Furthermore, research challenges in processing big data in IoT networks were discussed. Finally, a case study on fog data analytics in healthcare was presented, along with an experimental analysis using fog and cloud computing. The future work motivates researchers to carry out an in-depth review of current state-of-the-art techniques for adopting security in fog data analytics.

## Figures and Tables

**Table 3:** Comparison of authentication techniques.

Authors	Features	Auth Tiers	Control Agent	Pros	Cons
Banyal RK et al. [93]	Arithmetic-based CAPTCHA calculation, OTP, and IMEI-based authentication mechanism	3	Server	Resistance to multiple attack type	Computational complexity
Emam AHM [94]	Authentication through dynamic link sent on registered email address	2	Server	Cost-effective	Email address may get compromised
Kumar S et al. [95]	OTP entered through personal device	2	Client and Server	OTP needs to be entered using personal device	Access lost if registered Device is lost or damaged
Usman AA et al. [96]	Security token generated using preshared pin numbers, location, and time	2	Client and Server	Cost-effective	Clock synchronization problem
Liu S et al. [97]	Sends QR code to register mobile device’s Bluetooth address	2	Server	Cost-effective	Mutual authentication not considered
Ahmad S et al. [98]	Smart card and biometric scanning	3	Server	Multiple factors required to access services	Smart card mandatory
Singh TG et al. [56]	Based on graphical patterns	2	Server	User convenience	Patterns are predictable
Soni P et al. [99]	Splitting and distribution of OTP over different channels	3	Server	Difficult to listen covertly	User onconvenience
Dhamija A [100]	Requires hardware token	2	Server	Resistant to different types of attacks	Expensive solution

**Table 4:** Comparison of various works on orchestration.

Author	Network Architecture	Type	Unique Feature
Zaalouk et al. [111]	SDN-based	Orchestration-focused	Security-oriented, in charge of turning on/off applications that deal with security issues.
Mayoral et al. [108]			Migration of virtual machines between different network domains.
Vilalta et al. [112]			Hierarchical SDN architecture for heterogeneous wireless and optical networks. Also introduces end-to-end provisioning and recovery procedures in a multi-domain network.
Jaeger [110]	NFV-based		Focused on extending the European Telecommunications Standards Institute (ETSI) NFV reference architecture to manage and orchestrate security functions.
Furtado et al. [113]	-	Choreography-focused	Uses middleware for choreography able to automatically deploy and execute services. The middleware is also responsible for monitoring the service composition execution and for performing automatic resource provisioning and service reconfiguration to achieve agreed QoS levels.
Cherrier et al. [114]	SDN-based		Studies the impact of using orchestration and choreography in wireless sensor and actuator networks (WSANs) using mathematical analysis and also application experiments.
Velasquez et al. [107]	-	Hybrid approach	Uses orchestration along with choreography to achieve distributed as well as centralized management simultaneously.

**Table 5:** Comparison of resource-scheduling techniques.

Authors	Case Study	Algorithm Used	Performance Measurement	Pros	Cons
Static approaches					
Bitam et al. [118]	General	Bees life algorithm	CPU execution time, Allocated memory	-Managing allocated memory -Low CPU execution time	-Static scheduling -Low scalability
Fan et al. [83]	General	Ant colony optimization	Total profit, Guarantee Ratio	Maximizing profits of fog providers	High time complexity
Rahbari et al. [123]	EAHD application, Intelligent surveillance application	Symbiotic organisms search	Energy utilization, Network usage, Cost	-Minimizing energy utilization -Low execution cost	High execution time
Kabirzadeh et al. [125]	Intelligent surveillance application	Hyper-heuristic based	Energy consumption, Execution time, Network usage, Cost	-Minimizing energy consumption -Low cost and low time	Low scalability
Dynamic approaches					
Sun et al. [117]	Word count	NSGA-II	Service latency, Stability	-Low execution time -High scalability -Low latency	High cost
Cardellini et al. [119]	Word count, Log stream processing	Adaptive-based	Node utilization, Application latency, Inter-node traffic	-Enhancing runtime scheduling -Low Latency -Low execution time	-Low availability -Low scalability -Centralized topology
Zeng et al. [122]	Image Tasks	Heuristic-based	Task completion time	-Low computation complexity -Low response time	-High memory consumption
Chen et al. [67]	Vehicular cloud application	Heuristic-based	Response time, Queue length	-High dynamic efficiency -Using a formal method -Low time	Simple case study
Urgaonkar et al. [126]	Mobile application	Lyapunov optimization	Queue length, Cost	-Reducing state space -Performing a cost-optimal solution	-High cost -Low scalability
Hybrid approaches					
De Benedetti et al. [127]	Distributed robotics application	Adaptive-based	Scalability, Fault tolerance	-High interaction with IoT devices -Low latency -Low execution time	-Low scalability -High cost

**Table 6:** Comparison of latency.

Physical Topology	Latency (ms)	
	Cloud Layer	Fog Layer
Conf-1	221.32	56.32
Conf-2	248.91	64.20
Conf-3	282.45	66.12
Conf-4	1352.67	70.65
Conf-5	3452.77	90.78

**Table 7:** Comparison of network utilization.

Physical Topology	Network Utilization (KBs)	
	Cloud Layer	Fog Layer
Conf-1	151.23	40.43
Conf-2	168.43	43.95
Conf-3	334.78	51.56
Conf-4	890.67	108.37
Conf-5	1098.04	175.46

## Data Availability

No data are associated with this research work.
